# Markov chain-based impact analysis of the pandemic Covid-19 outbreak on global primary energy consumption mix

**DOI:** 10.1038/s41598-024-60125-3

**Published:** 2024-04-24

**Authors:** Hussaan Ahmad, Rehan Liaqat, Musaed Alhussein, Hafiz Abdul Muqeet, Khursheed Aurangzeb, Hafiz Muhammad Ashraf

**Affiliations:** 1 Department of Mechanical Engineering, University of Management and Technology, Sialkot Campus, Sialkot, 51310 Pakistan; 2 Department of Electrical Engineering and Technology, Government College University Faisalabad, Faisalabad, 38000 Pakistan; 3 Department of Computer Engineering, College of Computer and Information Sciences, King Saud University, P.O.Box 51178, Riyadh, 11543 Kingdom of Saudi Arabia; 4 Department of Electrical Engineering and Technology, Punjab Tianjin University of Technology, Lahore, Punjab Pakistan; 5 Department of Electrical and Computer Engineering, Sungkyunkwan University, Suwon, 16419 South Korea

**Keywords:** COVID-19 pandemic, Markov chain, Predictive modeling, Equilibrium state, Global primary energy consumption, Energy science and technology, Energy infrastructure

## Abstract

The historic evolution of global primary energy consumption (GPEC) mix, comprising of fossil (liquid petroleum, gaseous and coal fuels) and non-fossil (nuclear, hydro and other renewables) energy sources while highlighting the impact of the novel corona virus 2019 pandemic outbreak, has been examined through this study. GPEC data of 2005–2021 has been taken from the annually published reports by British Petroleum. The equilibrium state, a property of the classical predictive modeling based on Markov chain, is employed as an investigative tool. The pandemic outbreak has proved to be a blessing in disguise for global energy sector through, at least temporarily, reducing the burden on environment in terms of reducing demand for fossil energy sources. Some significant long term impacts of the pandemic occurred in second and third years (2021 and 2022) after its outbreak in 2019 rather than in first year (2020) like the penetration of other energy sources along with hydro and renewable ones in GPEC. Novelty of this research lies within the application of the equilibrium state feature of compositional Markov chain based prediction upon GPEC mix. The analysis into the past trends suggests the advancement towards a better global energy future comprising of cleaner fossil resources (mainly natural gas), along with nuclear, hydro and renewable ones in the long run.

## Introduction

The novel Coronavirus Disease 2019 (COVID-19) is a pandemic contagion whose first known case was reported in Wuhan, China, in December 2019^1^. Besides the health issues, the pandemic has surely affected the global society in various socio-economic dimensions like energy demand and supply^2–7^ economics^8^, climate and environment^9–12^ etc. as highlighted in many studies like to refer a few. The pandemic has resulted in an extensive uncertainty related to health and economy as asserted by^13^. The impact of the pandemic towards the pace of achievement of UN-defined sustainable development goals (SDGs) has been analysed by^14,15^. An exhaustive literature that analyses the multiple impact associated with the lockdown restrictions of pandemic on energy and power sector in terms of energy, climate change, rate of penetration of electric vehicles in the transport sector, alterations in supply and demand side, etc. has been presented by^16^. It comparatively analyses the pre- and post-pandemic situations from the view of points of fossil energy, economy, environment, and renewable energy future. This study is targeted at examining the global impact of the pandemic outbreak on the historical evolution of long term GPEC mix predicted in business-as-usual (BAU) fashion using Markov chain model.

The global primary energy consumption (GPEC) data employed in this study have been sourced from the annual reports published and archived by British Petroleum^17^. One of the objectives of such data collection is forecasting in BAU fashion^18^; as elaborated for the case of Chinese gas consumption structure by^19^. The considerations for the development of a cleaner low carbon future has been asserted by^20^ on global scale. Comparable study in China has been conducted by^21^ with focus on natural gas development. Primary energy, gas distribution and electricity generation mixes have been predictively modeled in many research efforts such as^22,23^. A lot of research covers the efforts in China to seek for an optimal energy mix like^24^. Combined oil and gas consumption forecast for Chinese economy has been carried out by^25^. The implications of rising shares of renewables in the Turkish primary energy structures have been analysed along with future roadmap for the purpose by^26,27^. Some energy related forecasting models in the available literature include shrikage estimator approach^28^, size distribution analysis^29^, hybrid dynamic model^30^; and neural networks^31^ etc.

Some research deals with the social, economic, technological, business, trade and environmental impacts of the pandemic COVID-19 on energy sector besides other sectors of economy. The global environmental impact in the form of lower carbon dioxide (CO_2_) emissions, owing to the lock-down due to the pandemic, has been deliberated by^9^. The influence of the pandemic on the electricity sector, along with relevant recommendations, has been put forth by^32^ in the global as well as the Indian viewpoints. The effect of pandemic on the international trade has been elaborated by^33^. The impact of pandemic on global energy sector and the way forward has been put forth in many studies like^2^. Comparable literature for Chinese energy sector are^34,35^. The concealed opportunities associated with the pandemic pertaining to the energy sector have been explained by^3,33^. The global effect of the pandemic on fossil fuel demand especially across major countries, has been studied by^36^ using a global vector auto regressive (GVAR) model. The analysis indicates that the pandemic along with economic lock-down will not lead to significant reduction in CO_2_ emissions in short time horizon of 2 years. Thus, the climate-change mitigation efforts should not be delayed due to the pandemic outbreak.

The relationship between pandemic shocks and fossil fuel supply market (gas, oil and coal) during first year of pandemic outbreak has been analysed by^37^ using a Markov switching approach. Markov chain approach performs well for foreseeing the percent shareholding patterns among stakeholders through using historical data. It has been used widely in available literature like for gas use structure prediction in China^19^, for prediction of total energy demand in China^22^, for gas demand structure prediction in Pakistan^38^, for primary energy and electricity demand structure prediction in Pakistan^39^, and for Chinese terminal energy structure forecasting^40^, to mention a few. The long run behaviour of Markov chains has been analysed by^41^ to be resulting in either periodic or steady state distributions. The compositional arrangement generally converges to a steady state, as a result of the classical predictive modeling based on combined use of Markov chain model and Chapman–Kolmogorov relation following^19^, after which there is no change in the percentage of shares among the stakeholders. Alternatively, the state of absolute equilibrium is achieved along the time series upon the occurrence of steady state. Markov chain may be regular or absorbing depending upon the nature of equilibrium distribution. In equilibrium state, the regular Markov chain results in the visible shares of all the shareholders while the absorbing Markov chain results in 100% share of one of the shareholders and zero share for the other ones.

The existing literature lacks assessment of the impact of the pandemic COVID-19 outbreak on global energy sector in terms of the Markovian equilibrium state of GPEC mix. This research tries to fill this gap through analyzing the historical GPEC data using Markovian equilibrium state. The answers to the following questions related to the global primary energy consumption (GPEC) have been strived to be explored through this study. First, in what way the GPE has been altering in the pre-pandemic era (investigation of the historical pre-pandemic data upto year 2018)? Second, is there any noticeable change in the historically evolving GPEC mix due to pandemic (analysis of GPEC mix pattern in the post-pandemic era, 2019 onwards)? Hence, the overall objective of the current study is the assessment of the impact of the pandemic COVID-19 on the GPEC mix in terms of historically evolving equilibrium state based on Markov chain model.

Markov chain model has been widely applied for the comparable problems of energy related compositional data analysis due to its capability of comprehensively understanding the past transformation of shareholding pattern (Pi graph) along discrete time series and storing this information in the form of successive one step and individual transition matrices. This information is later on used for prediction along the time series upto equilibrium state of shareholding pattern. Thus, any change in the past trends ultimately appears as a unique equilibrium shareholding pattern that has been subsequently proposed in this study as a tool for analysis of the potential impact of the pandemic outbreak on GPEC mix.

The contribution/novelty of this study is twofold. First, the equilibrium energy composition predicted using the classical approach is innovatively put forth as the ultimate outcome of the prevalent global primary energy strategy. Second, the Markov chain model (MCM) along with Chapman Kolmogorov approach (CKA) has been applied to explore the potential impact of the pandemic on the historical evolution of GPEC mix pattern. The investigative tool is classical, while its application is novel. The limitation of this study is the prediction of GPEC mix in business-as-usual scenario without considering the social, economic, environmental, technological and political constraints relevant to the global energy sector.

## Data and methods

The compositional time series data related to global GPEC are given in the empirical form in Table A1 in Appendix A. These data are briefly discussed as follows.

**Table 1:**
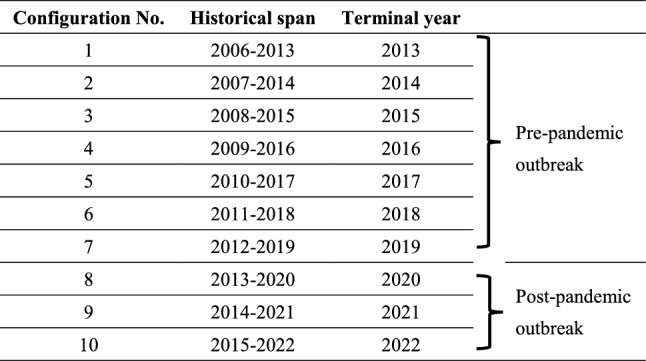
Configuration of historical data.

### Data and preliminary analysis

**Figure 1:**
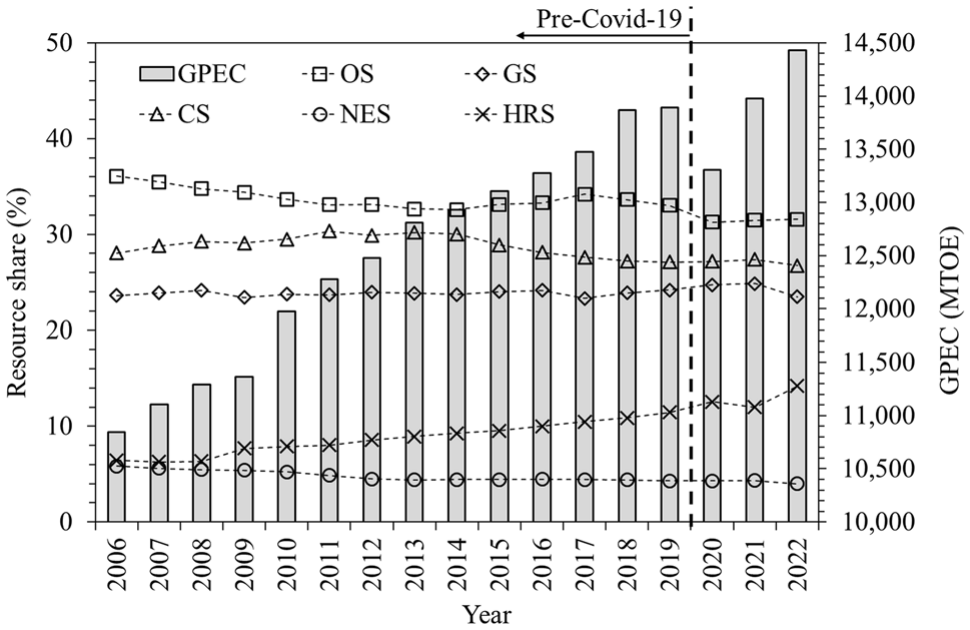
Plot of historical data of GPEC and corresponding resource shares.

The published data of past GPEC, during 2006–2022, have been plotted in Fig. 1 as columns scaled on the secondary axis as sourced from^17^. The various curves plot the compositional pattern among gaseous fuels share (GS), liquid fuels oils share (OS), coal fuel share (CS), nuclear electricity share (NS) and hydro/renewable electricity share (HRS).

#### Preliminary analysis into total energy consumption transition

**Figure 2:**
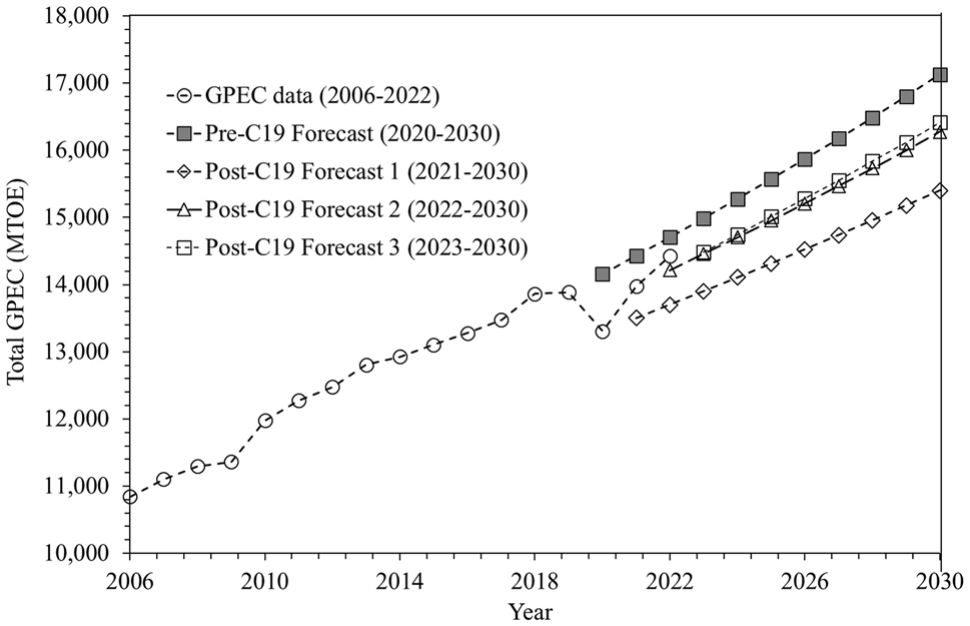
History and Prediction of GPEC in pre- and post-pandemic scenarios.

The GPEC values observed an increment of 10,537–13,865 MTOE at an annually compounded growth rate (ACGR) of 2.13% during pre-pandemic history of 2005–2018. However, a substantially reduced annual growth rate (AGR) of 0.17% has been witnessed during 2018–2019 followed by a negative and positive GR − 4.20% and 5.04% during 2019–2020 and 2020–2021 respectively which may be attributed to the temporarily reduced demand and its elasticity during the pandemic outbreak. The total GPEC has alternately increased and decreased from a value of 13,865 MTOE in 2018 to 13,889 MTOE, 13,306 and 13,976 during 2019–2021 respectively, thus observing alternate expansion and contraction of 0.17%, − 4.20% and 5.04% during the pandemic outbreak. The actual data of GPEC upto 2021, one pre pandemic forecast after 2019 along with three post pandemic forecasts, after one, two, and three years 2020, 2021 and 2022 respectively of its outbreak, have been plotted in Fig. 2.

In pre-pandemic scenario upto 2019, the total GPEC observed an ACGR of 1.92% during 2006–2019 leading to a GPEC estimate of 17,126 MTOE (717 exajoules (EJ)) by 2030. After, first year of pandemic outbreak in 2020, a negative AGR of − 4.20% was observed during 2019–2020 causing a drop of ACGR to 1.47% during 2006–2020 that led to a lower GPEC estimate of 15,401 MTOE (645 EJ) by 2030. Circumstances were somewhat restored after the second and third years 2021 and 2022 of the pandemic outbreak depicted by AGR values of 5.04% and 3.23% during 2020–2021 and 2021–2022, causing ACGR to rise to the values of 1.71% and 1.80% respectively. This led to a higher estimate of 16,275 MTOE (681 EJ) by 2030. Conclusively, the energy demand outlook has not yet been restored back to the pre-pandemic scenario as suggested by lower GPEC consumption forecast curves even after three years of its outbreak. This finding has been compared with those of other studies. According to^42^, in the pre- and post-pandemic scenarios in 2018 and 2020, demand forecasts for world’s fossil energy in 2050 are 456 and 424 exajoules (EJ) respectively. Hence, the pandemic reduces the global fossil energy demand forecast by 8% upto 2050. Other significant countries observed similar energy demand reductions^43^ like China, USA and European countries by 10.1%, 3% and range of 8–27% respectively in February 2020 compared to that in February 2019.

#### Preliminary analysis into historical GPEC mix transition

GPEC mix has observed pandemic induced transformation during 2019–2022 such that the expansion in HRS of 11.44–14.21% has been balanced by the contractions of OS, GS, CS and NS by 33.00–31.57%, 24.17–23.49%, 27.11–26.73% and 4.29–3.99% respectively. Thus the shares of cleaner hydro and renewable resources have observed increment as a favorable outcome of the pandemic outbreak. On the other hand, the liquid fuels, natural gas, coal and nuclear resources observed contracting shares trend after the pandemic outbreak. The pandemic outbreak has thus only a temporary impact on the shareholding pattern among the various energy resources. Shares and energy consumption values of gaseous, hydro and renewable resources have increased, an advantage that may be associated with the pandemic outbreak in line with the other global research efforts in this regard^11^.

#### Preliminary analysis into shares and growth rates of segregated fossil and non-fossil resources

**Figure 3:**
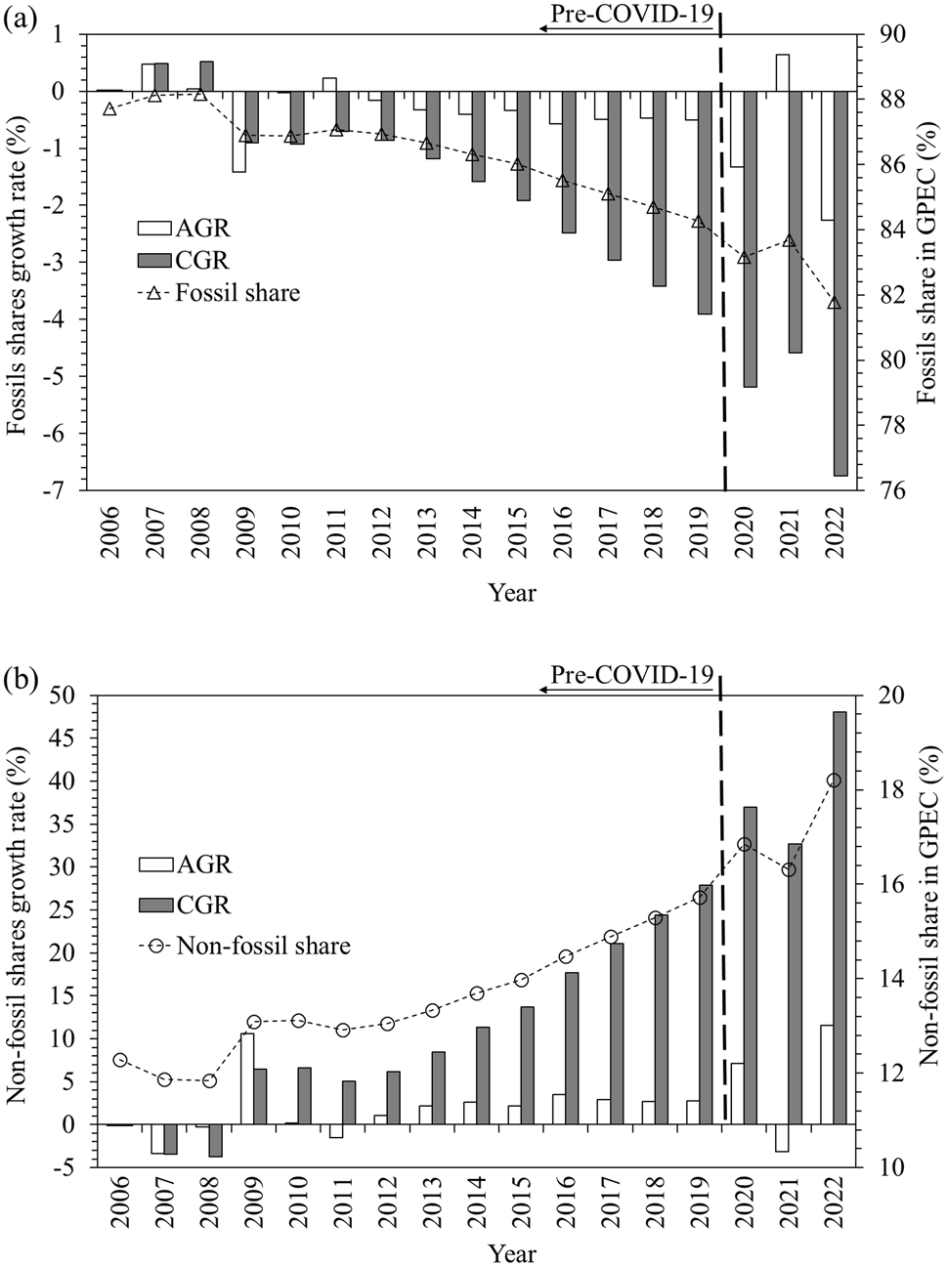
Shares and share growth rates of (**a**) fossil resources, (**b**) non-fossil resources.

Growth rates of shares of segregated fossil (OS, GS and CS) and non-fossil (NS and HRS) energy resources have been plotted in Fig. 3a and b respectively along with the respective resource shares shown as curve scaled on the secondary axis. The procedure for annual and collective shares growth rate calculation is given in Appendix C.

The fossil and non-fossil resources have mutually inverse trends of shareholding and growth rates. It is evident that shares of fossil and non-fossil observed negative and positive growth rates respectively, in the historical GPEC mix. Non-fossil share in GPEC recorded the highest AGR value of 11.59% in the post pandemic era during 2021–2022, the previous highest value was recorded to be 10.58% during 2008–2009. The pre- and post-pandemic average AGR values belonging to fossil energy shares have been − 0.31% and − 0.99% during 2006–2019 and 2020–2022 respectively. On the other hand, the pre- and post-pandemic average AGR values belonging to non-fossil energy shares have been 1.96% and 5.20% during 2006–2019 and 2020–2022 respectively.

Thus the pandemic initially expedited the advancement towards a cleaner and less fossil primary energy future for the globe, a condition that could be declared surely as a source of satisfaction for the humanity^44^ if continued. However, the impact of the pandemic was temporary demanding the continued efforts for climate mitigation in the long run according to the findings of^11,36,43^, etc.

### Method

**Figure 4:**
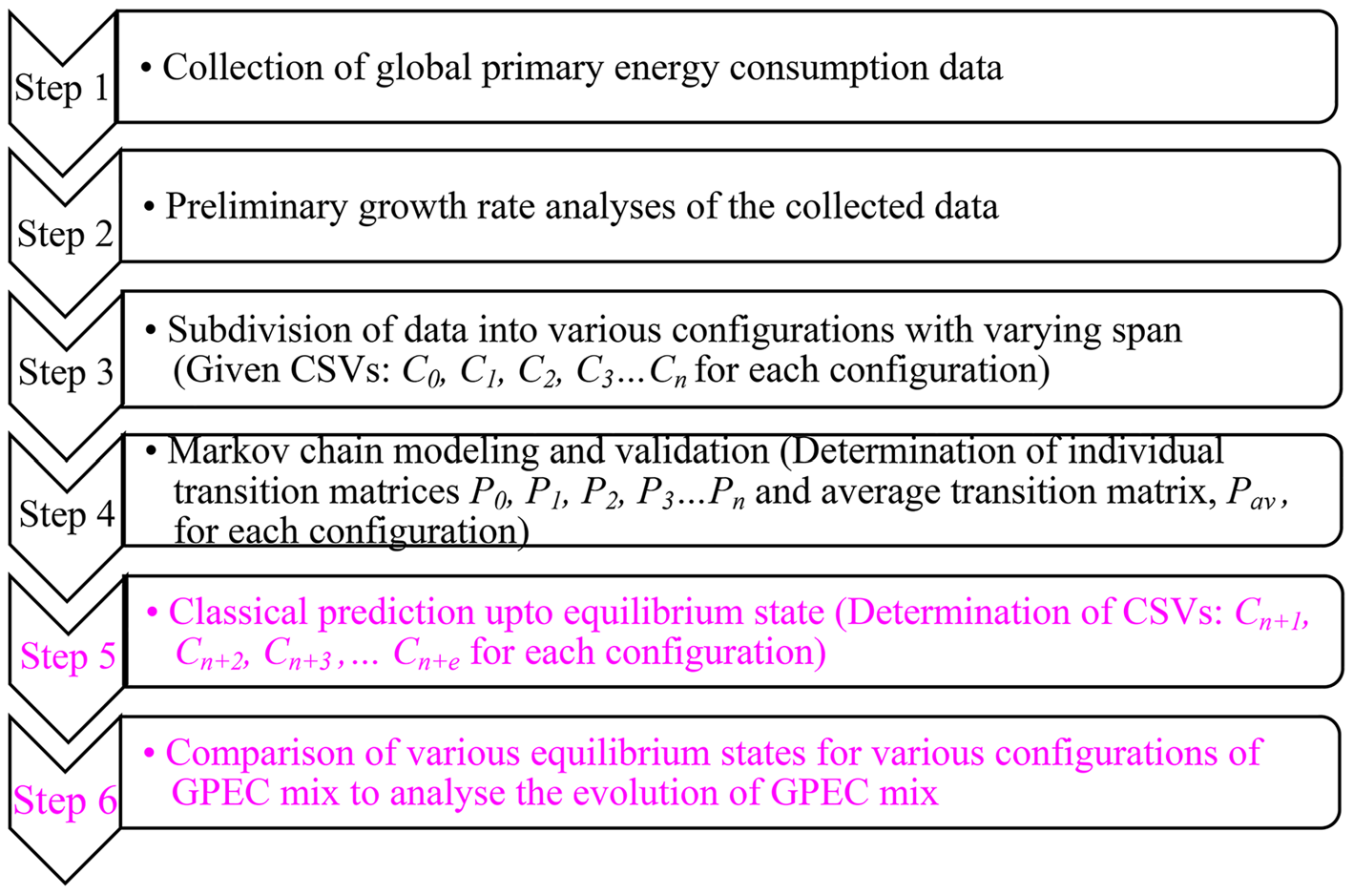
Method flow chart.

The method flow chart of this study is shown in Fig. 4.

This work begins with the collection of the GPEC data followed by its preliminary growth rate analyses. Steps 1 and 2 have already been discussed. After that, the data are subdivided into various configurations. The various configurations are then modeled as classical Markov chain and transition matrices are evaluated accordingly. The various configuration are projected using Chapman Kolmogorov approach^19^ upto equilibrium state. The equilibrium states associated with various configurations are finally compared to discuss the historical evolution of GPEC mix. The steps 3–5 are further elaborated as follows.

#### Data configuration

The available data of GPEC during 2006–2022^17^, have been subdivided into 10 latest historical configurations, 7 pre-pandemic and 3 post pandemic, as given in Table 1. Each of the 10 unique configurations contains the information of 8 consecutive yearly steps and thus 7 yearly transitions, 2006–2013, 2007–2014, 2008–2015…2015–2022 respectively. Configurations 1–7 are pre-pandemic outbreak so do not include its impact. Last three configurations include the impact of the pandemic outbreak so called post-pandemic configurations. There is a unit step increment in the spans of these configurations along the discrete time series (DTS).

#### Model

The past GPEC pattern can be modelled as Markov chain process along discrete time series summarized as follows. Assume that Xn (n ≥ 0) is an arbitrary process with the state space of Sn (n ϵ N); the arbitrary process can be called a Markov chain if the probability of (1) is applicable to all the cases of P{X_o_ = i_o_, X_1_ = i_1_, … , X_n_ = i_n_} > 0 as i_o_, i_1_, …, i_n_ , i_n+1_ ϵ S; i.e., given the past state transitions from the starting state at moment 0 to the final state at moment n, the next state at the moment n + 1 depends on the current state of moment n.

1 $$P \{\left.{X}_{n+1}= {i}_{n+1}\right|{X}_{0} = {i}_{0}, {X}_{1} = {i}_{1},... {,X}_{n}= {i}_{n}\} = P \{\left.{X}_{n+1}= {i}_{n+1}\right|{X}_{n} = {i}_{n}\}$$

A combination of Markov chain model (MCM) and Chapman-Kolmogorov approach (CKA) has been used for the predictive modeling and validation for various data configurations. More details may please be referred to^19,35,40,45,46^. The MCM captures the trend of the past prevailing policies, in the BAU fashion without considering the policy constraints, in terms of successive individual one step transition matrices, *P*_*0*_*, P*_*1*_*, P*_*2*_*, P*_*3*_*…P*_*n*_ and an average one step transition matrix (ATM), *P*_*av*,_ distinct for each configuration. In this way, ten different ATMs are calculated for ten different data configurations. The validity of ATMs is verified for the various configurations through the estimation of respective terminal state vectors and then comparing them with the actual terminal state vectors. After model validation, CKA is used to project the trend captured by MCM through a desired depth into the time series.

Given a sequence of past composition state vectors (CSVs) *C*_*0*_*, C*_*1*_*, C*_*2*_*…C*_*n*_, n ϵ N (the set of positive integers) belonging to a distinct configuration from initial to terminal years, CKA approach uses the terminal CSV, *C*_*n*_ along with P_av_, for projection along time series. The past data projection along the time series using CKA is continued until the attainment of equilibrium/steady state assumed as a quantitative means of analyzing the historical evolution in this study. The prediction of successive CSVs, in BAU fashion, is given by (2) for an i^th^ general CSV,* i* number of steps ahead of historical terminal state upto equilibrium state for a certain configuration.

2 $$ \begin{array}{*{20}c}    {C_{{n + 1}}  = } & {P_{{av}}  \cdot C_{n}  = } & {[S\left( {n + 1} \right)_{1} } & {S\left( {n + 1} \right)_{2}  \ldots } & {S\left( {n + 1} \right)_{q}  \ldots } & {S\left( {n + 1} \right)_{w} ]}  \\    {C_{{n + 2}}  = } & {P_{{av}}  \cdot C_{{n + 1}}  = } & {[S\left( {n + 2} \right)_{1} } & {S\left( {n + 2} \right)_{2}  \ldots } & {S\left( {n + 2} \right)_{q}  \ldots } & {S\left( {n + 2} \right)_{w} ]}  \\    {C_{{n + 3}}  = } & {P_{{av}}  \cdot C_{{n + 2}}  = } & {[S\left( {n + 3} \right)_{1} } & {S\left( {n + 3} \right)_{2}  \ldots } & {S\left( {n + 3} \right)_{q}  \ldots } & {S\left( {n + 3} \right)_{w} ]}  \\     \cdot  & \cdot & \cdot & \cdot & \cdot & \cdot  \\    \cdot & \cdot & \cdot & \cdot & \cdot & \cdot  \\    {C_{{n + i}}  = } & {P_{{av}}  \cdot C_{{n + i - 1}}  = } & {[S\left( {n + i} \right)_{1} } & {S\left( {n + i} \right)_{2}  \ldots } & {S\left( {n + i} \right)_{q}  \ldots } & {S\left( {n + i} \right)_{w} ]}  \\   \end{array}  $$

where, 1 ≤ i ≤ e ϵ N and subscript e symbolizes serial number of equilibrium state. The long range prediction into the time series results in monopolistic shareholding trend (MST) where one of the shareholders nullifies others by absorbing 100% shares thus making the study of historical evolution a senseless job. So, to put the compositional prediction into context, shares are predicted through some finite number of discrete steps along DTS. The finite number is taken as 100 in this study, so the predicted CSV hundred steps ahead of historical terminal state in BAU fashion, for a certain configuration, is given by (3).

3 $${C}_{n+100}={P}_{av} . {C}_{n+99}=[\begin{array}{cccc}{S\left(n+100\right)}_{1}& {S\left(n+100\right)}_{2}& \dots & {S\left(n+100\right)}_{q}\end{array}{\dots S\left(n+100\right)}_{w}]$$

where, *S* stands for an element in the CSV and the subscript q stands for the serial number of the general element in a general CSV, such that 1 ≤ q ≤ w ϵ N, w is the total number of shareholding elements in a general CSV. The subscript w has a value of 5 due to five shareholding energy resources in this study. The projected CSVs are distinct upto the equilibrium state, an inequality condition given in (4).

4 $${C}_{n+1}\ne {C}_{n+2}\ne {C}_{n+3}\ne . . . {C}_{n+i}\ne . . . {C}_{n+e}$$

where, subscript e symbolizes serial position of equilibrium state, 1 ≤ i ≤ e ϵ N. The successive states’ projection may be extended further ahead of absolute compositional equilibrium (ACE) as in (5) for kth CSV ahead of historical terminal state for a certain configuration.

5 $$  \begin{array}{*{20}c}    {C_{{n + e + 1}} } &  =  & {P_{{av}}  \cdot C_{{n + e}} }  \\    {C_{{n + e + 2}} } &  =  & {P_{{av}}  \cdot C_{{n + e + 1}} }  \\    {C_{{n + e + 3}} } &  =  & {P_{{av}}  \cdot C_{{n + e + 2}} }  \\     \cdot & \cdot & \cdot  \\    \cdot & \cdot & \cdot  \\    {C_{{n + k}} } &  =  & {P_{{av}}  \cdot C_{{n + k - 1}} }  \\    \cdot & \cdot & \cdot  \\    \cdot & \cdot & \cdot  \\    {C_{{n + \infty }} } &  =  & {P_{{av}}  \cdot C_{{n + \infty  - 1}} }  \\   \end{array}  $$

where, (e + 1) ≤ k ≤ ∞ ϵ N. Ahead of the equilibrium state, the CSV no more change along time series, an equality condition termed as the stationarity of MCM given in (6).

6 $${C}_{n+e}= {C}_{n+e+1}= {C}_{n+e+2}={C}_{n+e+3}\dots = {C}_{n+k}\dots ={C}_{n+\infty }$$

## Results and discussion

### Markov chain modeling and validation results

The ten ATMs attained through the application of compositional Markov chain model for the ten configurations have been attached as Table B in Appendix B. The modeling is validated by reproducing terminal state vector for each configuration followed by evaluation of mean absolute percentage error (MAPE) across the five actual and estimated elements. MAPE values for various configurations have been listed in Table 2.

**Table 2:**
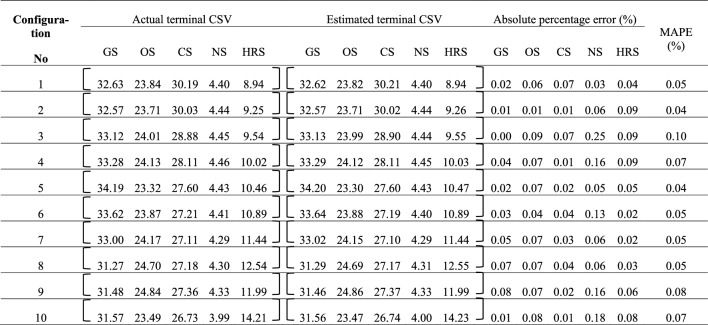
Validation of Markov chain model for various configurations.

The various share values in the actual and estimated terminal CSVs are limited to two decimal places, so reader should not be confused with invisible difference between the elements. The mean modeling error varies from 0.04 to 0.10% across the various configurations. This error is quite acceptable thus validating the utility of the Markov chain model and ten ATMs applied for the purpose of long term prediction. After validating ATMs, subsequent projection of the various configurations upto their respective equilibrium states satisfactorily is discussed in next section.

### Prediction of GPEC mix

**Figure 5:**
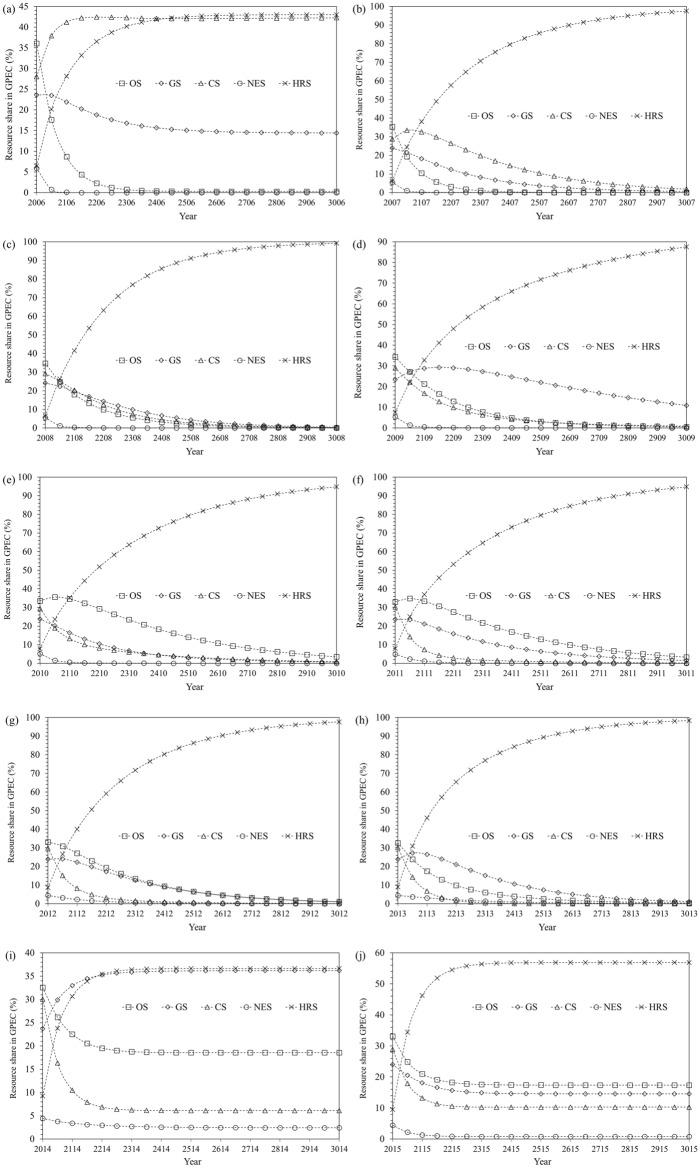
(**a**) to (**j**). Historical evolution of long term prediction of GPEC mix.

Results of the analysis of the historical evolution of GPEC have been discussed in this section. The long term equilibrium projections of GPEC state vectors, in BAU fashion, against the ten past configurations are shown in Fig. 5a to j, thousand years ahead of the historical terminal states specific to each configuration, respectively.

It is worth noting at this stage that the selection of 1000 years ahead prediction span is just random to show the trend of approaching equilibrium for which the share curves become absolutely horizontal. This theoretical state has been used for the mutual comparison of the historically evolving state of GPEC mix. The initial eight configurations suggest the exclusive role of HRS as given in Fig. 5a to h with the long term equilibrium share values of 43.01%, 100%, 100%, 99.55%, 99.99%, 99.99%, 100% and 100% respectively. However, the GPEC mix has been restored to its pre-pandemic situation of first configuration with equilibrium HRS shares of 36.67% and 56.92% respectively as shown by Fig. 5i and j for the configurations 9 and 10 respectively. The configuration 1 demonstrated a regular Markov chain trend with visible shares of other energy resources along with HRS. The configurations 2–8 demonstrated an absorbing Markov chain trend with exclusive share of HRS in long run GPEC mix. The configurations 9 and 10 including the impact of the pandemic, however, demonstrated a regular Markov chain trend in the long run again in line with configuration 1. Thus the pandemic outbreak reverted the absorbing Markov chain trend of GPEC mix in the long run.

As suggested by the forecast 2, 2 years after the pandemic outbreak, the fossil energy resources come out as the vital ones with collective equilibrium share of 60.92% in the long run. Split shares of fossil fuels are 36.22%, 18.58% and 6.12% for gas, oil and coal respectively. However, the forecast 3, 3 years after the pandemic outbreak, suggests the way of GPEC mix towards the pre-pandemic scenario, with major share 57.76% of non-fossil fuels. Thus the pandemic suggests the importance of conventional fossil energy resources along with the non-fossil ones especially HRS for the development of cleaner energy future. This finding is supported by the argument that before the pandemic outbreak, the development of renewable energy resources was expeditiously underway due to enhancing affordability as presented by^47^.

**Figure 6:**
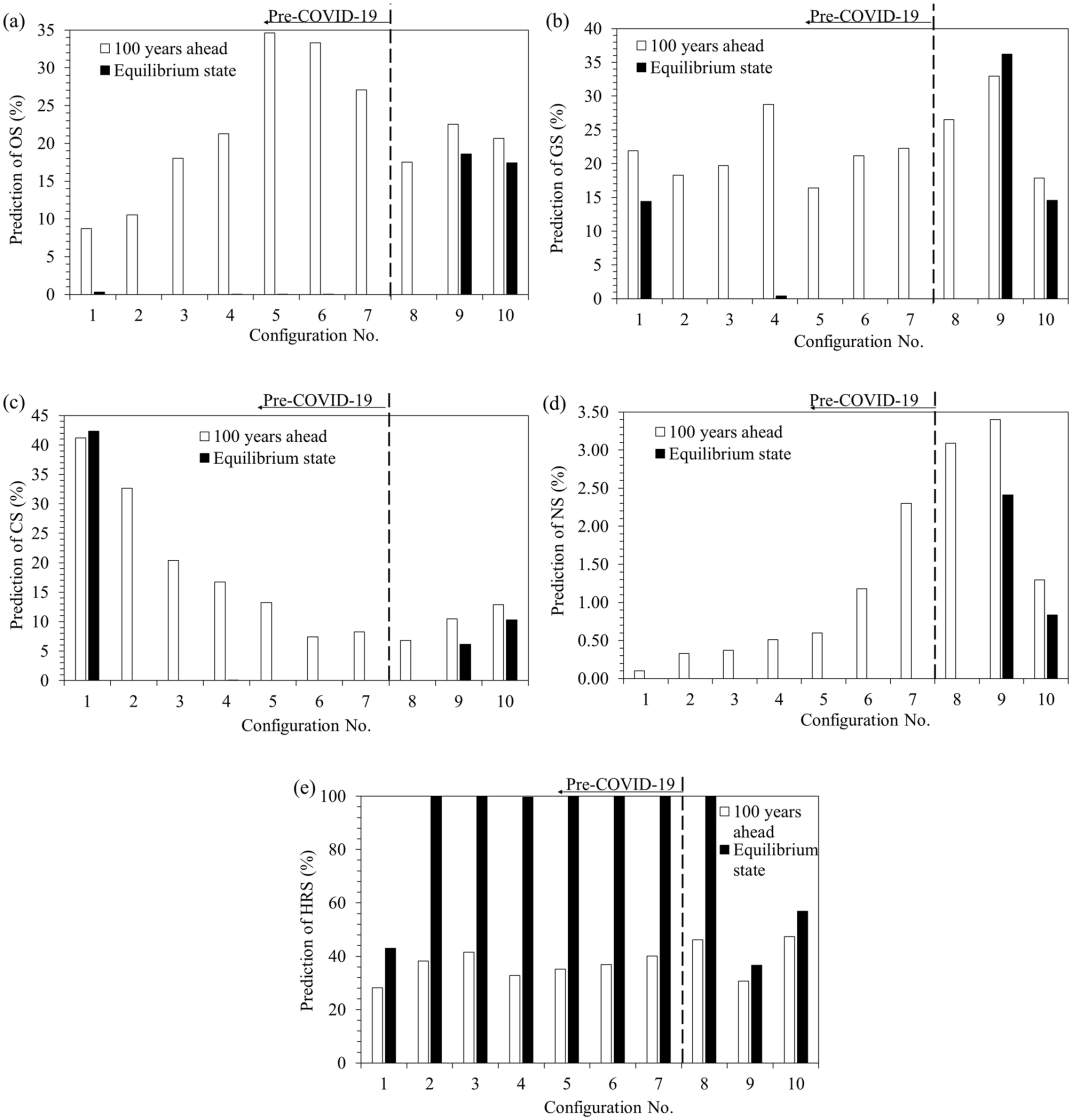
Predicted shares hundred years ahead and in ACE state for: (**a**) OS (**b**) GS (**c**) CS (**d**) NS (**e**) HRS.

The long term prediction into the time series results in MST where one of the shareholders nullifies others by absorbing nearly 100% shares thus making the analysis of historical evolution a senseless job. So, to put the shares prediction into the context of historical evolution, CSV predicted 100 years ahead of historical terminal states in BAU fashion, has been assumed for comparison of various configurations for studying historical evolution of GPEC mix. It is worth noting here that the choice of 100 years is purely random for comparison of prediction trends among the various data configurations only, and some other comparable value can be used instead for the same purpose. The CSVs resulting from the predictive modeling of the various data configurations, both 100 years ahead state and the ACE state, for various data configuration are plotted against historical terminal years, in Fig. 6a to e for oil, gas, coal, nuclear and hydro/renewable resources respectively.

The prediction results of two types of historically evolving states of GPEC composition, first, the ACE state, and second hundred years ahead of the terminal year, are separately discussed as follows.

#### Historical evolution of long term GPEC mix

The historical evolution of the projected ACE state initially suggested CS as the major resource for the configuration 1. The Markov chain then became absorbing one while the HRS assumed MST for the configurations 2–8. The pandemic had initially no significant impact on the historical evolution of GPEC in the long run except for expediting the advancement towards a cleaner future upto 2020. However, composition is restored to far behind the pre-pandemic era of 2013 with significant shares of both fossil and non-fossil resources, the only difference is the replacement of CS with GS in post-pandemic scenario. HRS will be the most significant resources in the long run to the advantage of the humanity. This projection suggests the ultimate sustainable development across the globe in terms of green energy utilization. Therefore, the earth is going to be a better place for its inhabitants compared with the history in terms of the lower air pollution^20,48^. NS, in spite of being the smallest one, seems expanding the most perpetually along DTS. The greater-than-null long term shares of GS, OS, CS and NS, amounting 36.22%, 18.58%, 6.12% and 2.41% respectively in descending order, have been induced in the second year 2021 after the pandemic outbreak before which HRS continued observing MST. The GPEC mix is on the way to be expeditiously restoring to the pre-pandemic scenario with major HRS value of 56.92% accompanied by OS, GS, CS and NS values of 17.39%, 14.57%, 10.28% and 0.84% respectively. Thus, the post-pandemic developments favour the simultaneous utilization of cleaner hydro and renewable resources along with the fossil fuels in the long run.

#### Historical evolution of 100 years ahead GPEC mix

The historical evolution of the 100 year ahead projection suggests the expanding shares pattern for the OS, NS and HRS through 8.69–20.67%, 0.10–1.29% and 28.13–47.306% respectively while contracting shares pattern for GS and CS through 21.90–17.87% and 41.19–12.87% in predicted GPEC mix from configurations 1–10. The contracting shares trend of OS during 2017–2019 was initially continued upto the first year but reverted in the second year of the pandemic outbreak. Likewise, a perpetual contraction trend of CS 41.19–8.26% during 2012–2019 continued in the first year but reversed in the second and third years of the pandemic outbreak. The perpetual expansion trend of NS has not got changed upto second year of the pandemic shock. However, it observed contraction in third year after pandemic outbreak. Thus, the pandemic outbreak induces the development of a low carbon future skewed towards the cleaner renewable energy resources alongside the fossil fuels.

HRS will be the most significant global energy resource after a hundred years in the long run to the advantage of the humanity. The shares of more carbon emitting fossil fuels are on the way towards significant contraction. On one hand, the pandemic outbreak has induced the reversal of upward mobility of OS as well as the continuation of the negative growth rate in CS. On the other hand, the expansions in GS, NS and HRS have been further expedited by the pandemic in the first year after its outbreak. Thus the pandemic accelerated the advancement towards a cleaner and more sustainable future upto 2020, as explored by^15^. However, circumstances changed to some extent in 2021 when MST of HRS was compensated by other resources shares, thus demanding continuation of efforts for climate mitigation and cleaner energy future^36^. COVID-19 has proved to be obviously a temporarily occurring event with no significant long term impact on the upward mobility of renewable energy shares in GPEC. After restoration of GPEC mix to that in pre-pandemic scenario, there will be a more severe concern in renewable energy future in post-pandemic scenario. Cost curtailment, enhanced efficiency and carbon regulations will expedite the renewables’ shares enhancement in post-pandemic era as asserted by^11^.

Prominent research belonging to the topic of impact of COVID-19 pandemic outbreak on energy structure is presented by^35^. It takes into account the Chinese energy mix data 2011–2019 comprising of coal, oil, gas and renewable energy resources, and projects it 41 discrete yearly steps ahead upto 2060 in BAU fashion using MCM. For the Chinese economy, the shares of coal will contract from 56.32 to 21.31% during 2020–2060. On the other hand, shares of gas, oil and renewable resources will expand from 8.46–17.50%, 19.26–28.45% and 15.96–32.74% respectively.

### Resource shares and their growth rates in predicted GPEC mix

**Figure 7:**
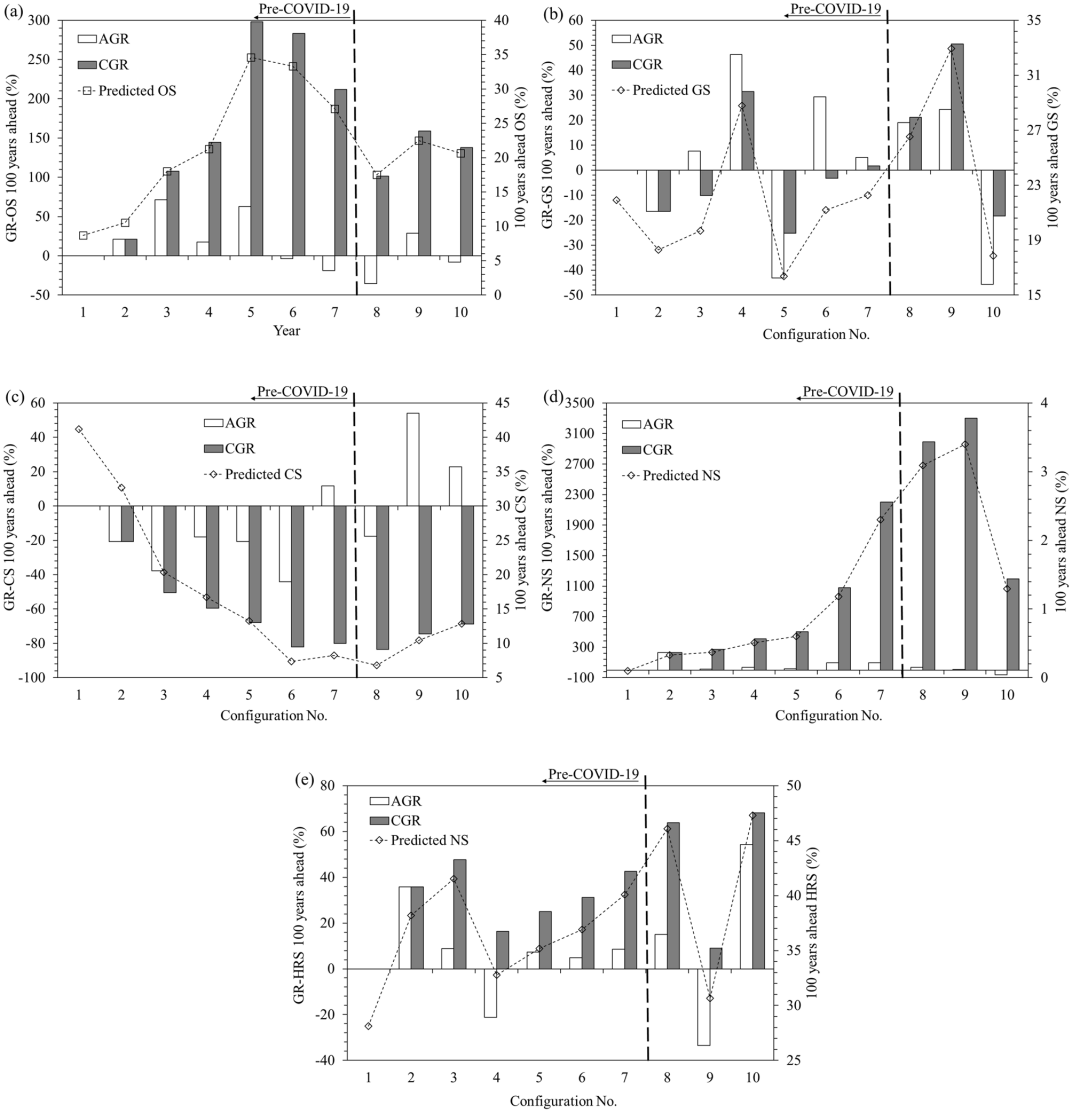
Hundred year ahead predictive evolution of resource shares and their growth rates for: (**a**) OS (**b**) GS (**c**) CS (**d**) NS (**e**) HRS.

The hundred years ahead predicted shares in BAU fashion, along with annual growth rate (AGR) and collective growth rates (CGR) values, have been plotted in Fig. 7a to e for the five energy resources respectively.

OS has demonstrated an inverted V-type shareholding trend along DTS, with some negative and many positive AGR values. The largest and smallest ever AGR values of 71.5% and -35.4% were observed for terminal years of 2015 and 2020 respectively. GS has demonstrated a W-type shares trend, with some negative and many positive AGR values. The largest and smallest ever AGR values of 46.3% and − 43.1% were observed for terminal years of 2016 and 2017 respectively. CS has demonstrated a nearly perpetual downhill-type shareholding trend, with many negative and a few positive AGR values. The smallest and largest ever AGR values of − 44.2% and 53.9% have been observed for 2018 and 2021 respectively. NS, despite of being the smallest shareholder, has demonstrated a nearly perpetual uphill-type shareholding trend, with many negative and a few positive AGR values. The smallest and largest ever AGR values of − 44.2% and 53.9% have been observed for 2018 and 2021 respectively. HRS has demonstrated an alternate up and down-hill type shareholding trend, with alternative positive and some negative AGR values. The largest and smallest ever AGR values of 35.7% and − 33.5% have been observed for 2014 and 2021 respectively. The impact of the pandemic shock occurred in the second post-pandemic year 2021 as reversal of long prevailing expansion and contraction trends of HRS and OS depicted by AGR values changing from − 35.4% to 28.7% and 15.0% to − 33.5% respectively. Terminal CGR values observed by OS, GS, CS, NS and HRS are 137%, 40%, − 74%, 2991% and 16% respectively in the hundred years ahead prediction upto 2021.

The inverted V-type, W-type, downhill-type, uphill-type and up and down hill type predicted shareholding trends for OS, GS, CS, NS and HRS respectively are independent of the prediction span, number of years ahead of the terminal year. This has been demonstrated by Figs. D1 and D2 in Appendix D, for instance for ten and twenty years ahead of the terminal states along DTS. So, any comparable choice other than hundred years ahead can similarly work for the prediction analysis.

## Concluding remarks

The evolution of the global energy sector has been modeled in this research in terms of the equilibrium state, a characteristic of the classical Markov chain model, to analyze the potential impact of COVID-19 pandemic outbreak on GPEC mix. Alternatively, the equilibrium state attribute of the classical compositional Markov chain model has been innovatively applied in this research to analyze the impact of the pandemic COVID-19 outbreak on the evolution of global primary energy mix. The modeling error in this study is limited to within 0.10%, quite within the acceptable limit. The major contribution of and the literature gap filled by this study is the utilization of equilibrium state feature of Markov chain model to track the impact of the pandemic COVID-19 shock on the GPEC mix. The key concluding remarks are:

The GPEC values observed an ACGR of 2.15% during pre-pandemic history of 2004–2018, reduced to a value of 0.17% during post-pandemic year 2018–2019 followed by a negative value of − 4.20% during 2019–2020. This reduction may be attributed to the reduced demand during the pandemic outbreak. This may be termed as a natural reset. Thus the pandemic, despite of being apparently detrimental, has initially come out as a blessing in disguise through inducing, at least temporarily, a significant reduction in the fossil fuels demand as well as the related environmental pollution loading.

The pandemic initially expedited the MST on part of HRS to become an unaided primary energy source in the long run. The pandemic outbreak leads to the development of a cleaner energy future comprising gas, oil, coal and nuclear resources besides renewable ones, as suggested by the equilibrium shares of 36.21%, 18.58%, 6.12%, 2.42% and 36.67% respectively determined by this study.

Some significant impacts of the pandemic shock were not observed in the first year but in the second and third years 2021 and 2022 after its outbreak like: the long prevailing MST on part of HRS came to an end thus sparing some room for other resources as well, and the reversal of long prevailing pre-pandemic trends of expansion and contraction on part of HRS and OS respectively.

NS despite of being the smallest shareholder in GPEC has demonstrated the largest CGR value of 1191.95% during 2013–2022.

The restoration to the pre-pandemic circumstances in second and third years after its outbreak suggests that the global energy demand will be simultaneously met by both fossil and non-fossil resources in the long run. This suggests the development of an energy future based mainly on renewable resources in the upcoming decades.

This study analysed the impact of pandemic on historical evolution of equilibrium state of GPEC in BAU scenario with a disadvantage of not taking into account the social, economic, environmental, technological and political issues to be faced. Such analyses may become a scope of further study. This study may be used as a guide to map the impact of the pandemic and alike shocks for other compositional data of percentage shares to deal with in practice, beyond energy, like those related with production, economics, business, sales, marketing, trade, supply chain, agriculture and planning, etc.

### Supplementary Information

Supplementary Information.

## Data Availability

The data analysed in this work has been taken from publically available and free-to-access internet resource at link https://www.bp.com/en/global/corporate/energy-economics/statistical-review-of-world-energy.html.

## Abbreviations

ACE: Absolute compositional equilibrium
AGR: Annual growth rate
ATM: Average one step transition matrix
BAU: Business-as-usual
CKA: Chapman–Kolmogorov approach
CS: Coal share
DTS: Discrete time series
GR: Growth rate
GS: Gas share
HRS: Hydroelectricity and renewables share
MST: Monopolistic shareholding trend
NS: Nuclear share
SDGs: Sustainable development goals
ACGR: Annually compounded growth rate
AHP: Analytic hierarchy process
CGR: Collective growth rates
COVID: Coronavirus disease
CSV: Composition state vector
EJ: Exa joule
GPEC: Global primary energy consumption
MCM: Markov chain model
MTOE: Million tons of oil equivalent
OS: Oil share
UN: United Nations
