# Author Correction: Markov chain-based impact analysis of the pandemic Covid-19 outbreak on global primary energy consumption mix

**DOI:** 10.1038/s41598-024-61288-9

**Published:** 2024-05-07

**Authors:** Hussaan Ahmad, Rehan Liaqat, Musaed Alhussein, Hafiz Abdul Muqeet, Khursheed Aurangzeb, Hafiz Muhammad Ashraf

**Affiliations:** 1 Department of Mechanical Engineering, University of Management and Technology, Sialkot Campus, Sialkot, 51310 Pakistan; 2 Department of Electrical Engineering and Technology, Government College University Faisalabad, Faisalabad, 38000 Pakistan; 3 Department of Computer Engineering, College of Computer and Information Sciences, King Saud University, P.O.Box 51178, Riyadh, 11543 Kingdom of Saudi Arabia; 4 Department of Electrical Engineering and Technology, Punjab Tianjin University of Technology, Lahore, Punjab Pakistan; 5 Department of Electrical and Computer Engineering, Sungkyunkwan University, Suwon, 16419 South Korea

Correction to: *Scientific Reports* 10.1038/s41598-024-60125-3, published online 24 April 2024

The original version of this Article contained an error in the Funding section where the project number was incorrect.

“This Research is funded by Research Supporting Project Number (RSPD2023R553), King Saud University, Riyadh, Saudi Arabia.”

now reads:

“This research is funded by Researchers Supporting Project Number (RSPD2024R553), King Saud University, Riyadh, Saudi Arabia."

The original Article has been corrected.

