# Supplementary material for: Markov chain-based impact analysis of the pandemic Covid-19 outbreak on global primary energy consumption mix
Source: Sci Rep. 2024 Apr 24;14:9449. doi: 10.1038/s41598-024-60125-3 (PMC11043445; doi:10.1038/s41598-024-60125-3)
Supplement: Supplementary file 1 — Supplementary Information. [file 41598_2024_60125_MOESM1_ESM.docx]

# Appendix A

Table A1. Historical state vectors of global primary energy consumption mix

| Year | Global primary energy consumption mix | | | | |
| --- | --- | --- | --- | --- | --- |
|  | OS (%) | GS (%) | CS (%) | NS (%) | HRS (%) |
| 2006 | 36.07 | 23.59 | 28.05 | 5.86 | 6.43 |
| 2007 | 35.48 | 23.88 | 28.77 | 5.61 | 6.27 |
| 2008 | 34.78 | 24.14 | 29.25 | 5.49 | 6.35 |
| 2009 | 34.40 | 23.42 | 29.09 | 5.40 | 7.69 |
| 2010 | 33.66 | 23.74 | 29.49 | 5.23 | 7.88 |
| 2011 | 33.07 | 23.67 | 30.34 | 4.88 | 8.04 |
| 2012 | 33.11 | 23.94 | 29.90 | 4.49 | 8.56 |
| 2013 | 32.63 | 23.84 | 30.19 | 4.40 | 8.94 |
| 2014 | 32.57 | 23.71 | 30.03 | 4.44 | 9.25 |
| 2015 | 33.12 | 24.01 | 28.88 | 4.45 | 9.54 |
| 2016 | 33.28 | 24.13 | 28.11 | 4.46 | 10.02 |
| 2017 | 34.19 | 23.32 | 27.60 | 4.43 | 10.46 |
| 2018 | 33.62 | 23.87 | 27.21 | 4.41 | 10.89 |
| 2019 | 33.00 | 24.17 | 27.11 | 4.29 | 11.44 |
| 2020 | 31.27 | 24.70 | 27.18 | 4.30 | 12.54 |
| 2021 | 31.48 | 24.84 | 27.36 | 4.33 | 11.99 |
| 2022 | 31.57 | 23.49 | 26.73 | 3.99 | 14.21 |

# Appendix B

Table B1. Average unit step transition matrices (ATMs) for GPEC data configurations 1-10

| a- 2006-2013 | OS | GS | CS | NS | HRS |
| --- | --- | --- | --- | --- | --- |
| OS | 0.9856 | 0.0026 | 0.0077 | 0.0000 | 0.0041 |
| GS | 0.0000 | 0.9946 | 0.0006 | 0.0000 | 0.0048 |
| CS | 0.0001 | 0.0007 | 0.9971 | 0.0000 | 0.0021 |
| NS | 0.0005 | 0.0081 | 0.0179 | 0.9600 | 0.0135 |
| HRS | 0.0000 | 0.0011 | 0.0026 | 0.0000 | 0.9963 |
| b- 2007-2014 | OS | GS | CS | NS | HRS |
| OS | 0.9877 | 0.0020 | 0.0060 | 0.0000 | 0.0043 |
| GS | 0.0000 | 0.9939 | 0.0006 | 0.0001 | 0.0054 |
| CS | 0.0001 | 0.0007 | 0.9963 | 0.0001 | 0.0028 |
| NS | 0.0005 | 0.0063 | 0.0134 | 0.9661 | 0.0137 |
| HRS | 0.0000 | 0.0000 | 0.0000 | 0.0000 | 1.0000 |
| c- 2008-2015 | OS | GS | CS | NS | HRS |
| OS | 0.9906 | 0.0011 | 0.0043 | 0.0000 | 0.0040 |
| GS | 0.0000 | 0.9939 | 0.0006 | 0.0001 | 0.0054 |
| CS | 0.0028 | 0.0021 | 0.9908 | 0.0001 | 0.0042 |
| NS | 0.0006 | 0.0055 | 0.0114 | 0.9690 | 0.0135 |
| HRS | 0.0000 | 0.0000 | 0.0000 | 0.0000 | 1.0000 |
| d- 2009-2016 | OS | GS | CS | NS | HRS |
| OS | 0.9922 | 0.0011 | 0.0042 | 0.0000 | 0.0025 |
| GS | 0.0000 | 0.9982 | 0.0006 | 0.0001 | 0.0011 |
| CS | 0.0036 | 0.0027 | 0.9877 | 0.0002 | 0.0058 |
| NS | 0.0007 | 0.0055 | 0.0113 | 0.9711 | 0.0114 |
| HRS | 0.0000 | 0.0000 | 0.0000 | 0.0000 | 1.0000 |
| e- 2010-2017 | OS | GS | CS | NS | HRS |
| OS | 0.9871 | 0.0047 | 0.0003 | 0.0001 | 0.0078 |
| GS | 0.0031 | 0.9944 | 0.0000 | 0.0001 | 0.0024 |
| CS | 0.0050 | 0.0034 | 0.9847 | 0.0002 | 0.0067 |
| NS | 0.0006 | 0.0019 | 0.0000 | 0.9943 | 0.0032 |
| HRS | 0.0000 | 0.0000 | 0.0000 | 0.0000 | 1.0000 |

| f- 2011-2018 | OS | GS | CS | NS | HRS |
| --- | --- | --- | --- | --- | --- |
| OS | 0.9953 | 0.0014 | 0.0009 | 0.0000 | 0.0024 |
| GS | 0.0033 | 0.9937 | 0.0003 | 0.0001 | 0.0026 |
| CS | 0.0053 | 0.0039 | 0.9830 | 0.0002 | 0.0076 |
| NS | 0.0012 | 0.0042 | 0.0012 | 0.9836 | 0.0098 |
| HRS | 0.0000 | 0.0000 | 0.0000 | 0.0000 | 1.0000 |
| g- 2012-2019 | OS | GS | CS | NS | HRS |
| OS | 0.9927 | 0.0023 | 0.0009 | 0.0000 | 0.0041 |
| GS | 0.0032 | 0.9937 | 0.0003 | 0.0001 | 0.0027 |
| CS | 0.0052 | 0.0034 | 0.9846 | 0.0002 | 0.0066 |
| NS | 0.0006 | 0.0018 | 0.0012 | 0.9916 | 0.0048 |
| HRS | 0.0000 | 0.0000 | 0.0000 | 0.0000 | 1.0000 |
| h- 2013-2020 | OS | GS | CS | NS | HRS |
| OS | 0.9871 | 0.0047 | 0.0003 | 0.0001 | 0.0078 |
| GS | 0.0031 | 0.9944 | 0.0000 | 0.0001 | 0.0024 |
| CS | 0.0050 | 0.0034 | 0.9847 | 0.0002 | 0.0067 |
| NS | 0.0006 | 0.0019 | 0.0000 | 0.9943 | 0.0032 |
| HRS | 0.0000 | 0.0000 | 0.0000 | 0.0000 | 1.0000 |
| i- 2014-2021 | OS | GS | CS | NS | HRS |
| OS | 0.9873 | 0.0048 | 0.0004 | 0.0001 | 0.0074 |
| GS | 0.0031 | 0.9952 | 0.0000 | 0.0000 | 0.0017 |
| CS | 0.0050 | 0.0035 | 0.9856 | 0.0001 | 0.0058 |
| NS | 0.0006 | 0.0019 | 0.0000 | 0.9944 | 0.0031 |
| HRS | 0.0025 | 0.0016 | 0.0022 | 0.0003 | 0.9934 |
| j- 2015-2022 | OS | GS | CS | NS | HRS |
| OS | 0.9873 | 0.0046 | 0.0004 | 0.0001 | 0.0076 |
| GS | 0.0035 | 0.9872 | 0.0000 | 0.0000 | 0.0093 |
| CS | 0.0026 | 0.0019 | 0.9877 | 0.0001 | 0.0077 |
| NS | 0.0010 | 0.0018 | 0.0000 | 0.9831 | 0.0141 |
| HRS | 0.0025 | 0.0015 | 0.0021 | 0.0002 | 0.9937 |

# Appendix C

The annual and collective growth rates of shares for an energy resource, *AGR* and *CGR*, are given by (C_1_) and (C_2_) respectively.

$AGR=\left( S_{i+1}- S_{i} \right)\times100/S_{i}$ (C_1_)

$CGR=\left( S_{i}- S_{0} \right)\times100/S_{0}$ (C_2_)

Where, 0 ≤ i ≤ n, n stands for the serial number of the historical terminal state for a configuration

*S_0_ =* Resource share in initial year (%) (1990 in current case)

*S_i_* = Resource share in i^th^ year (%)

*S_i+1_* = Resource share in (i+1)^th^ year (%)

# Appendix D


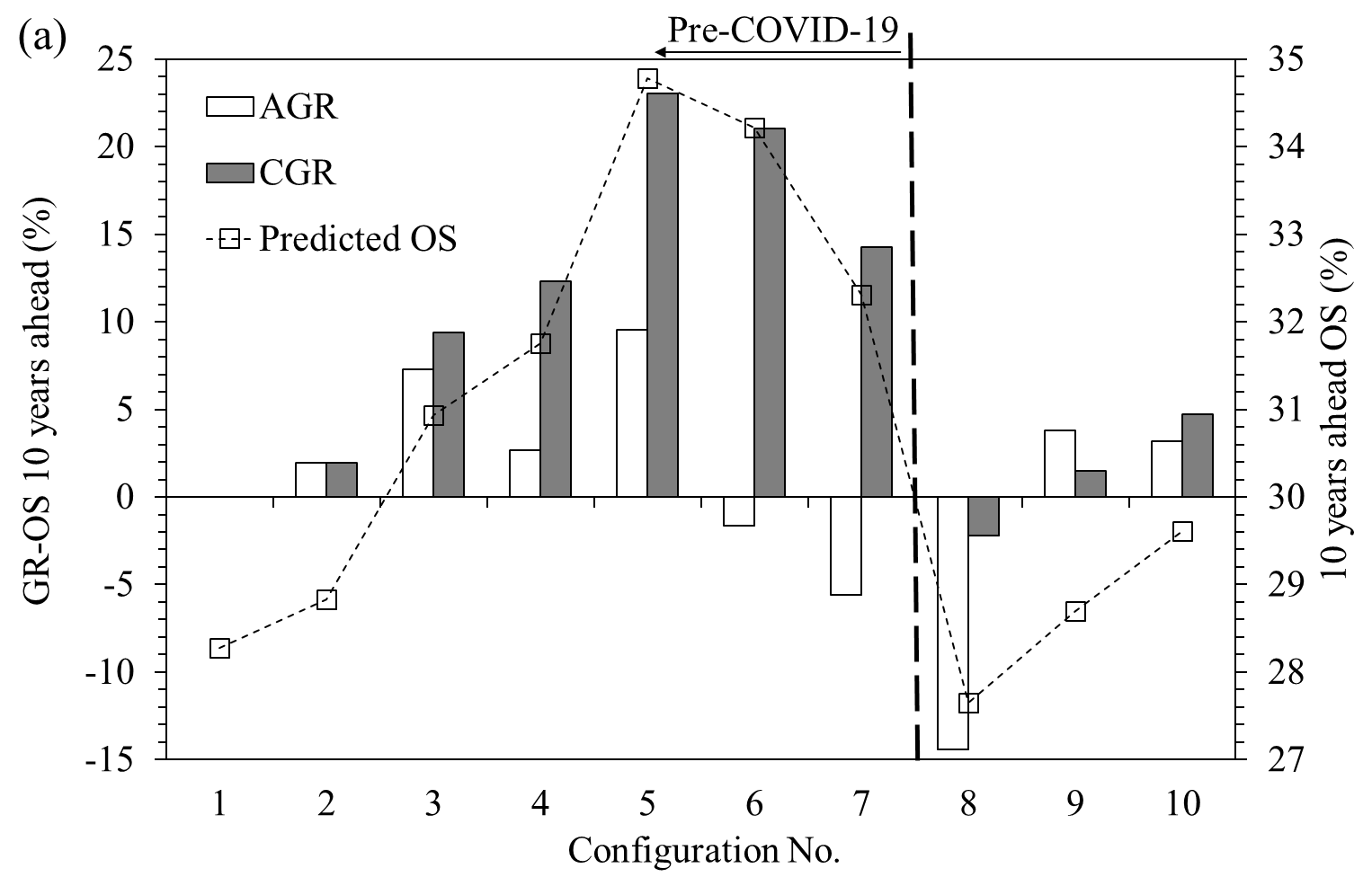

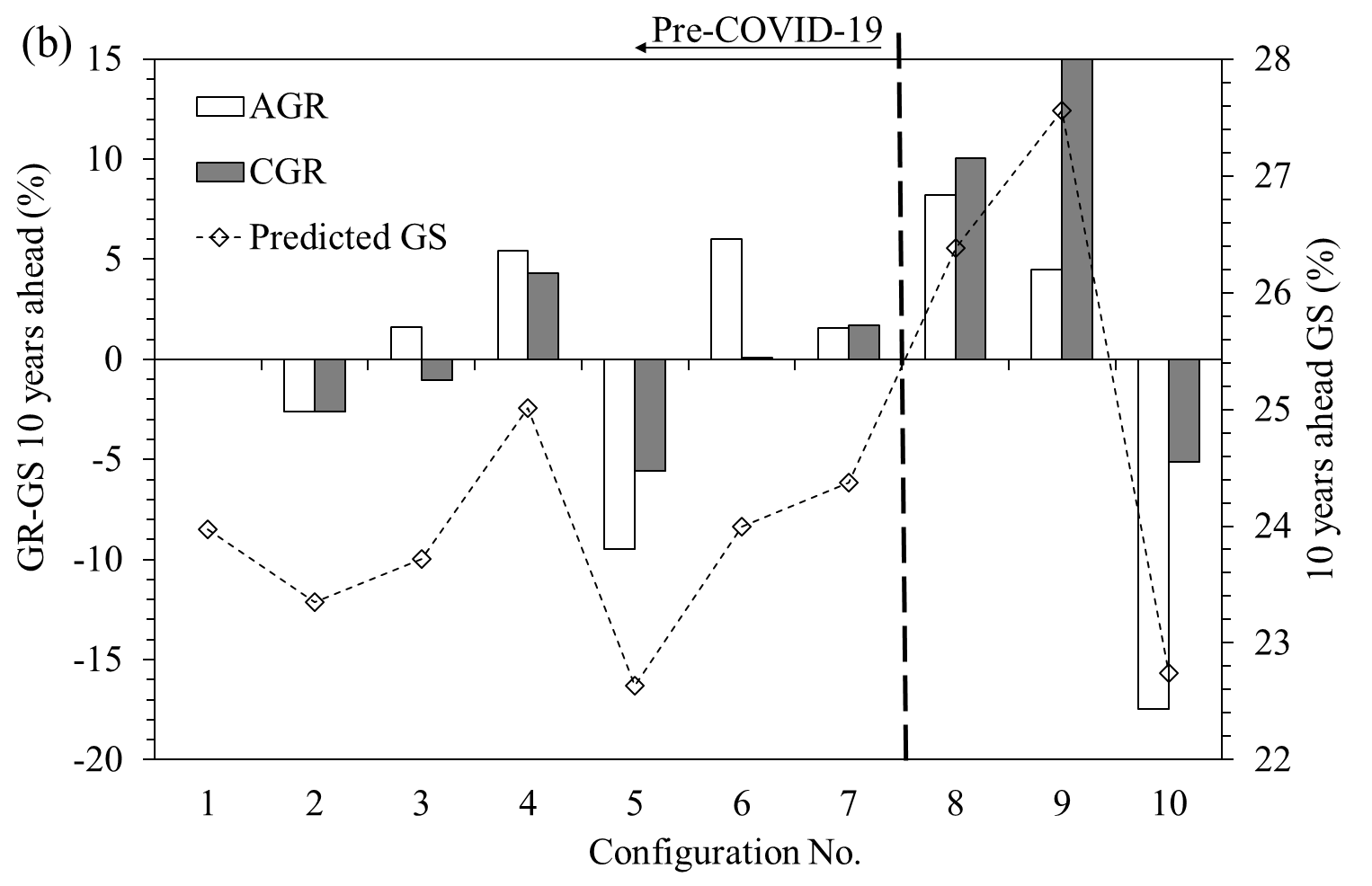

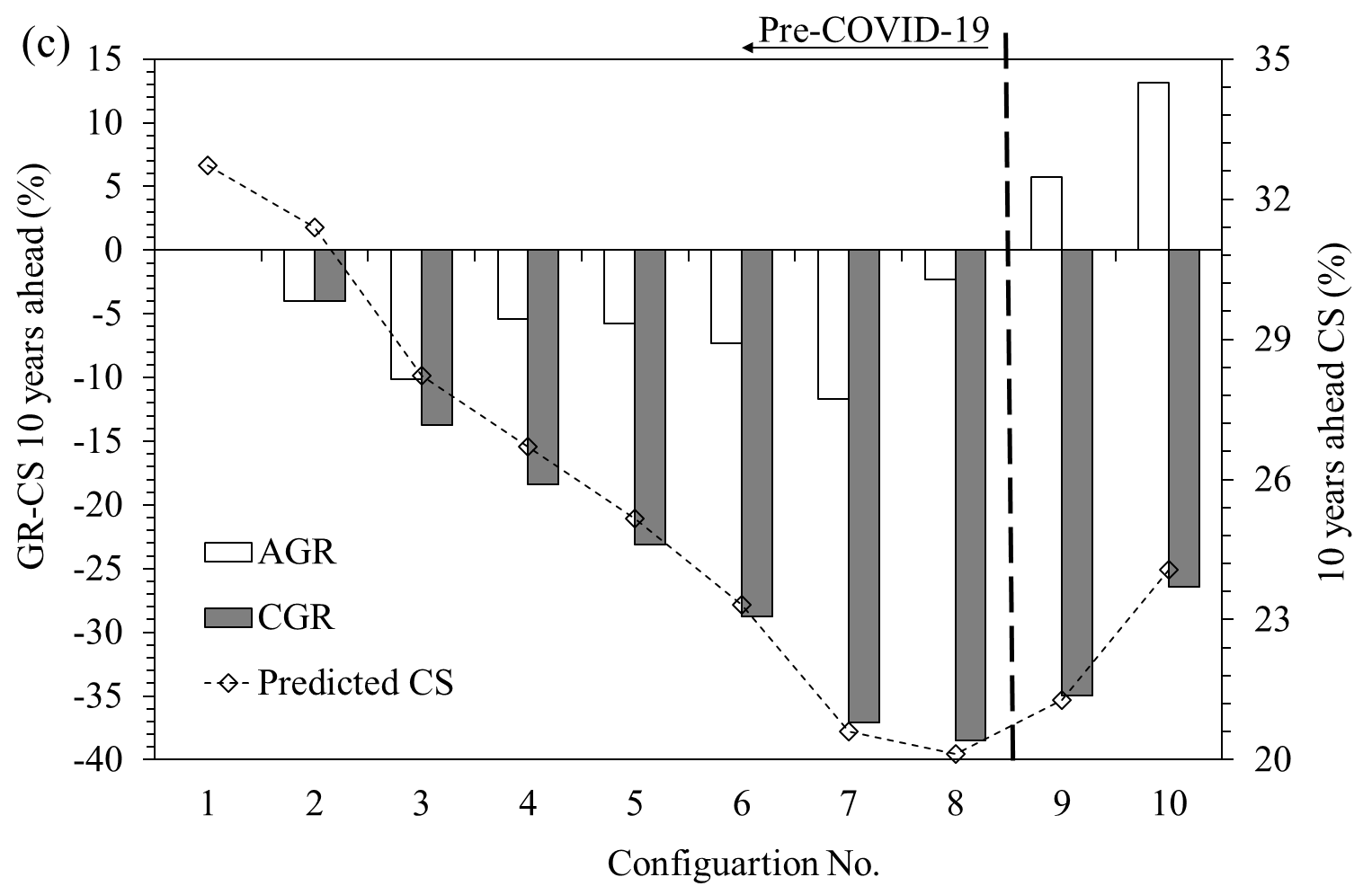

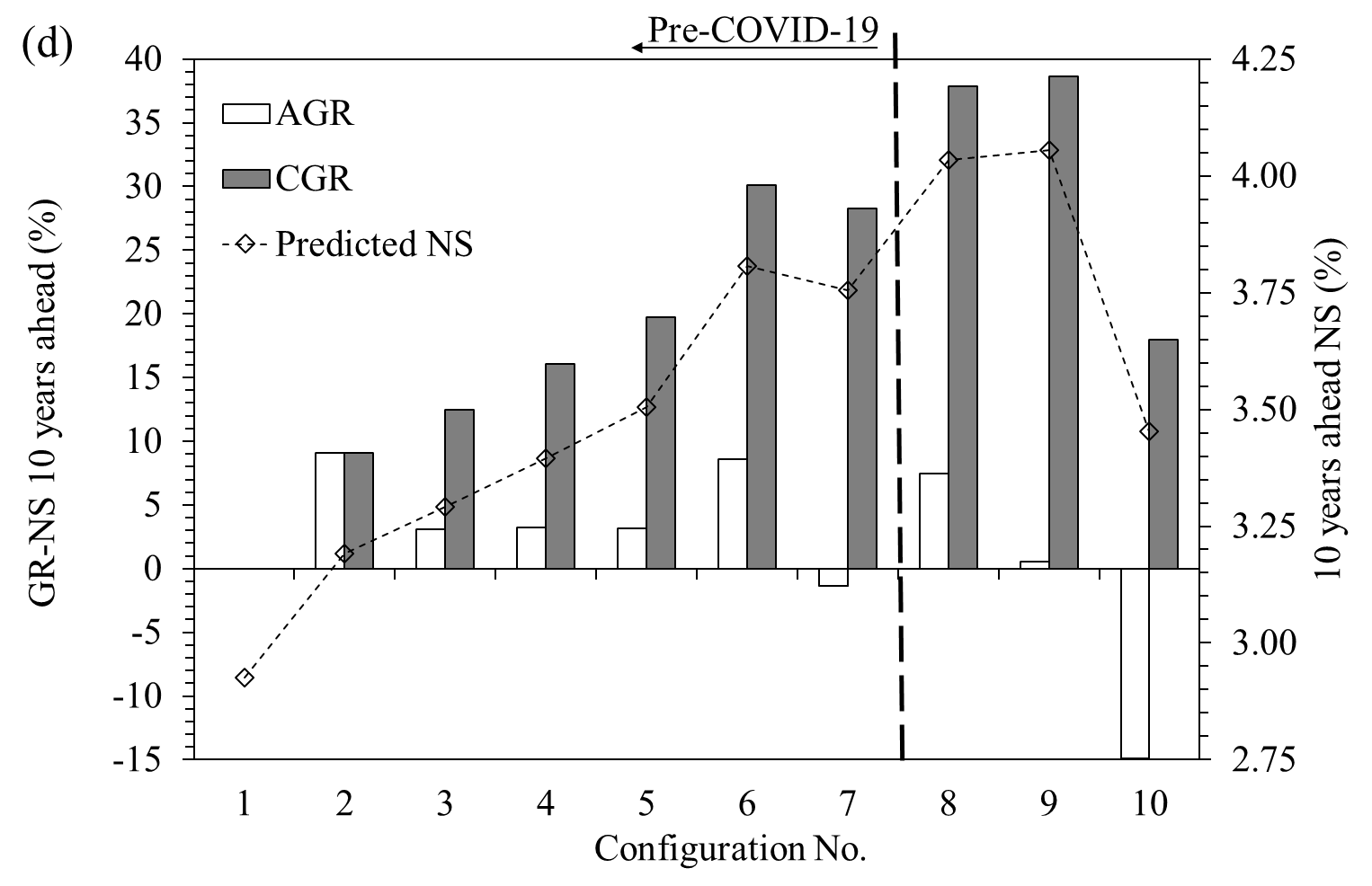

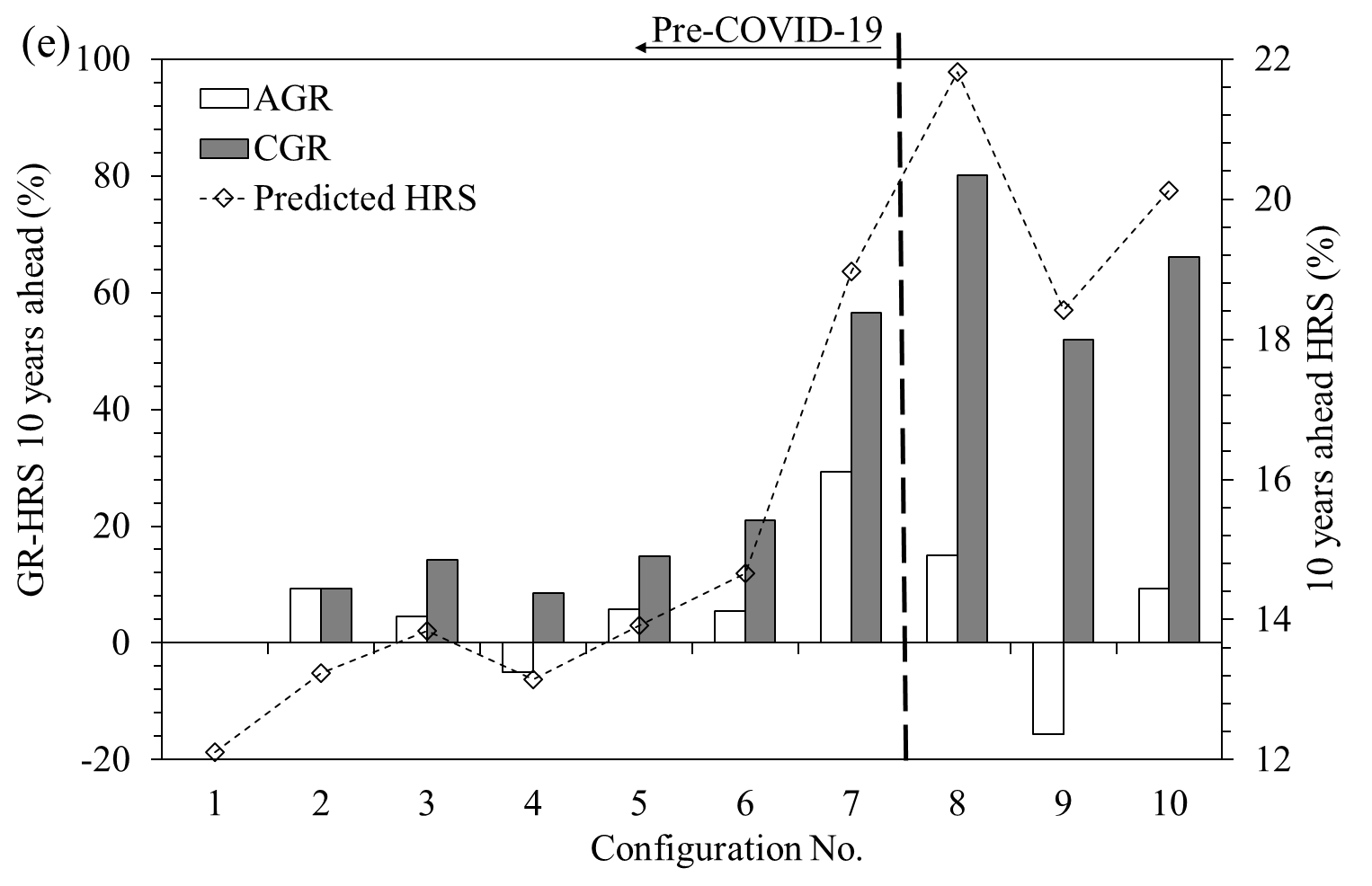


Figure D1. Ten year ahead predictive evolution of resource shares and their growth rates for: (a) OS (b) GS (c) CS (d) NS (e) HRS.


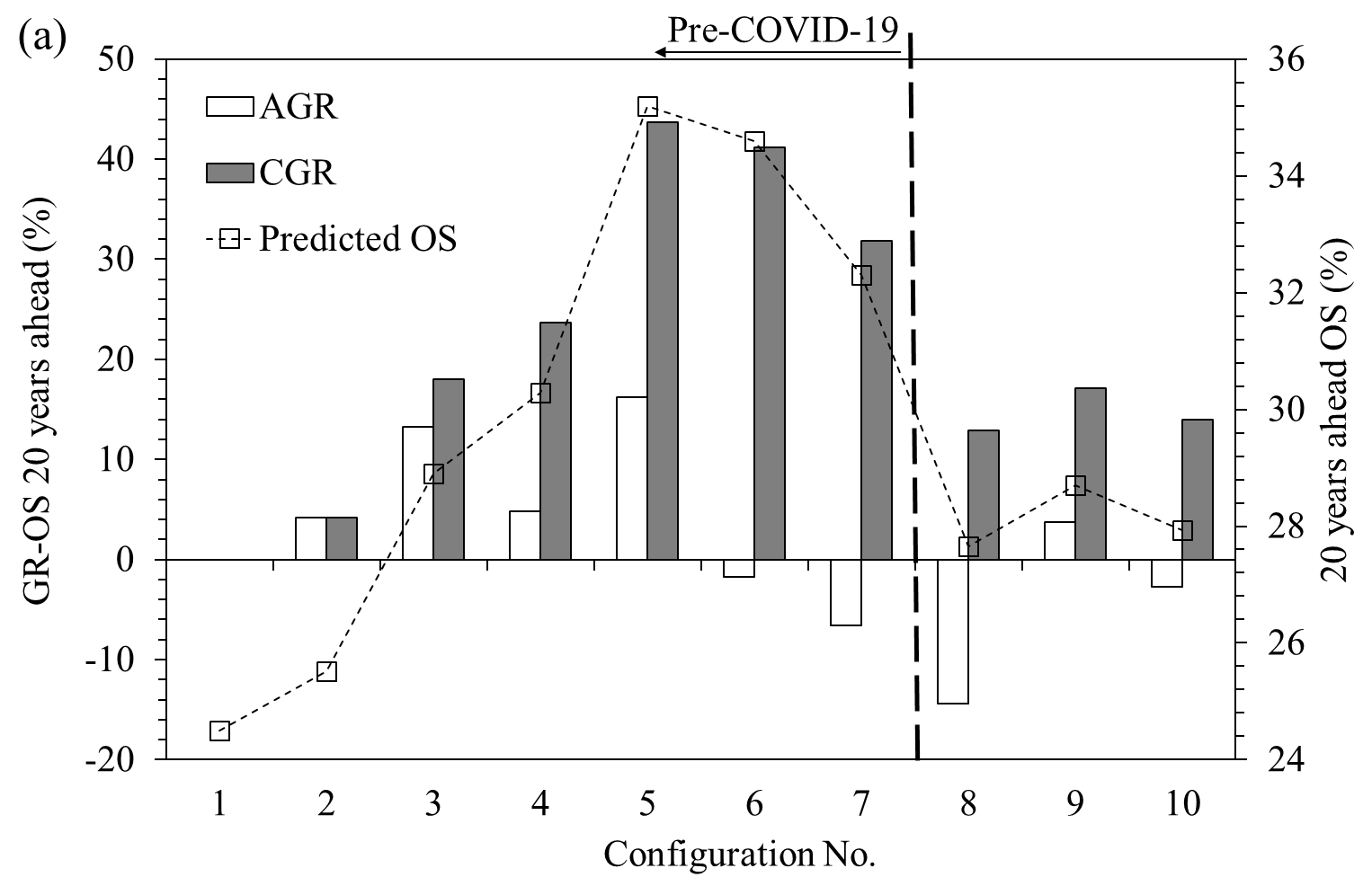

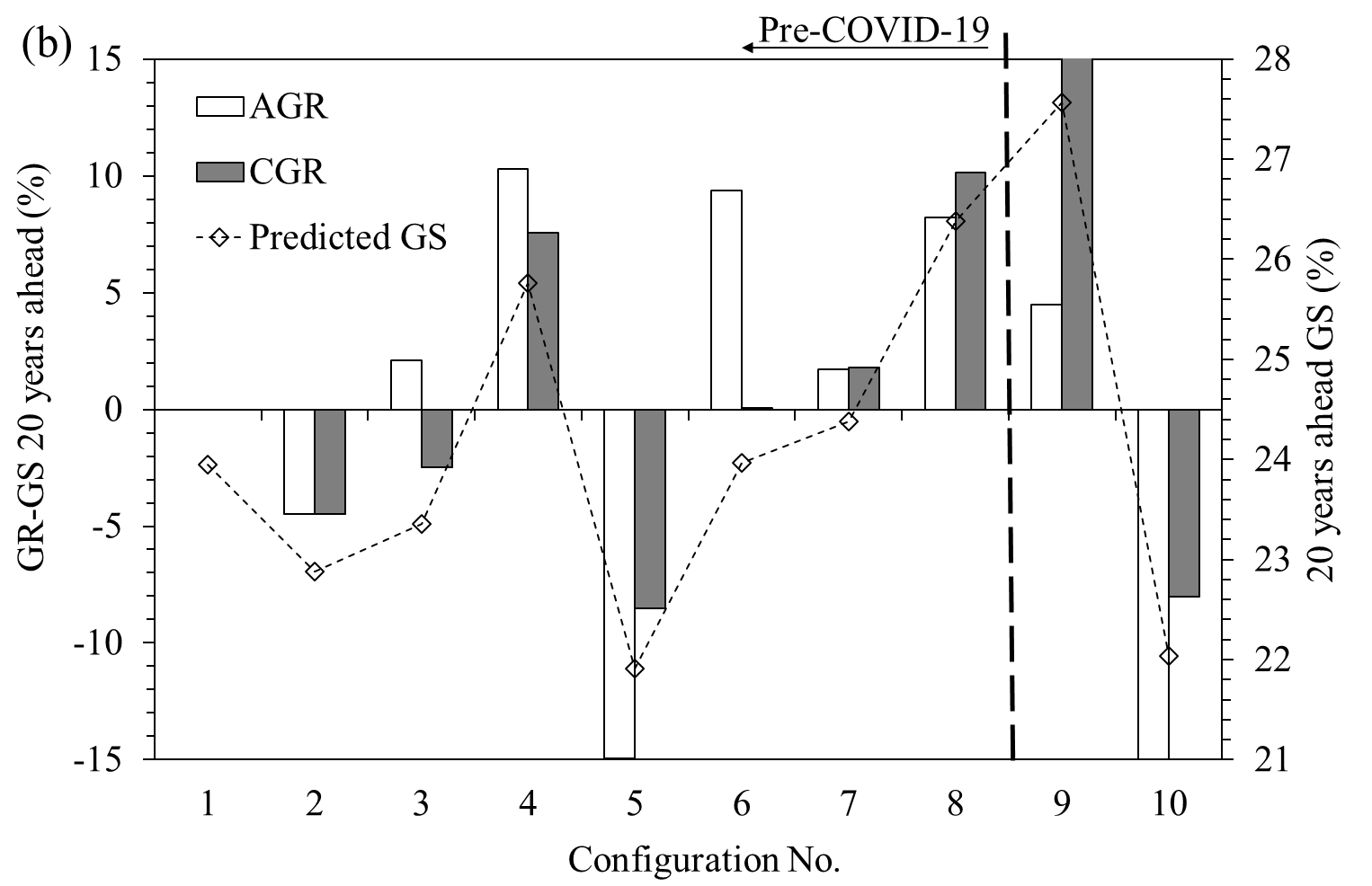

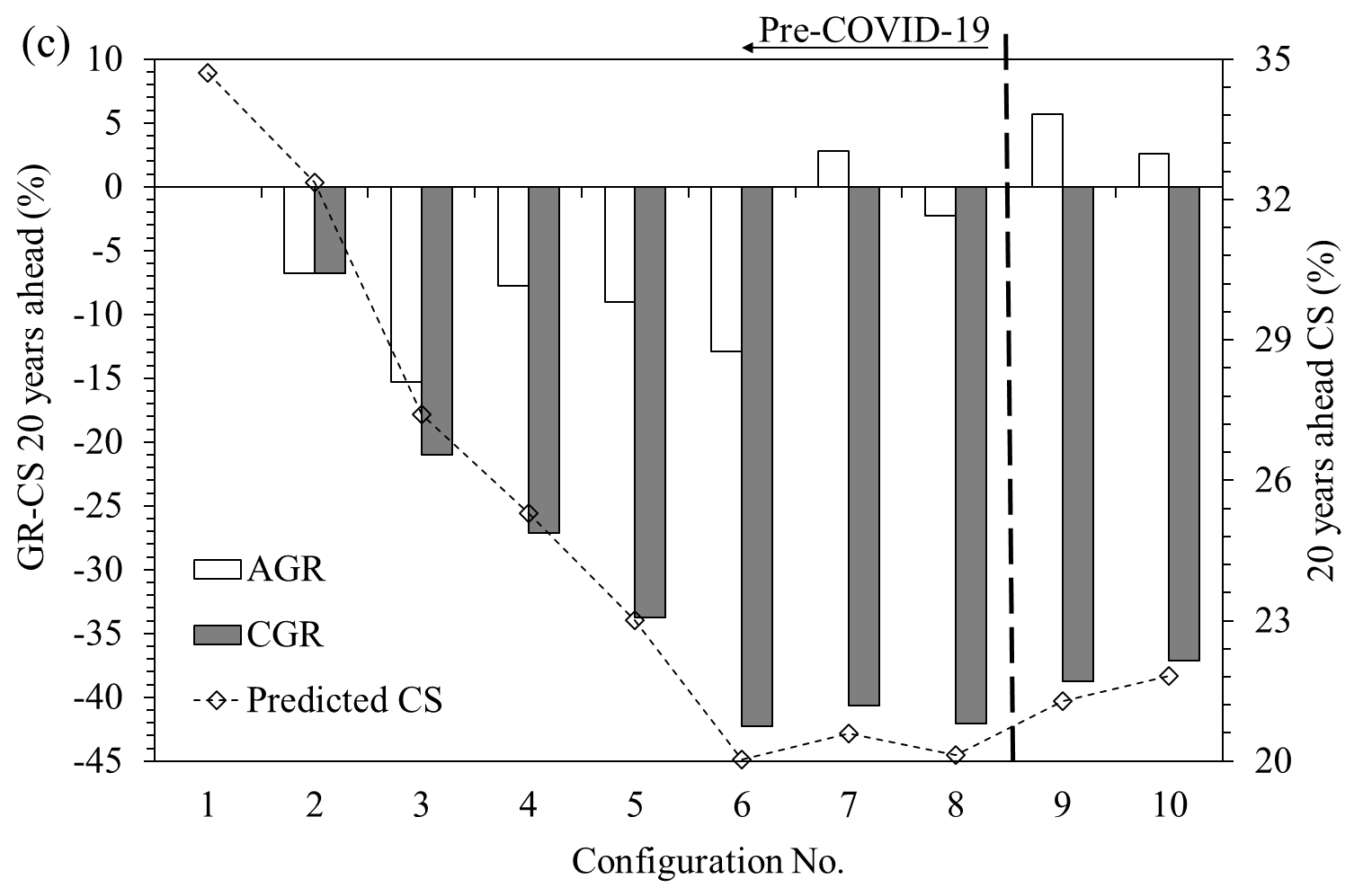

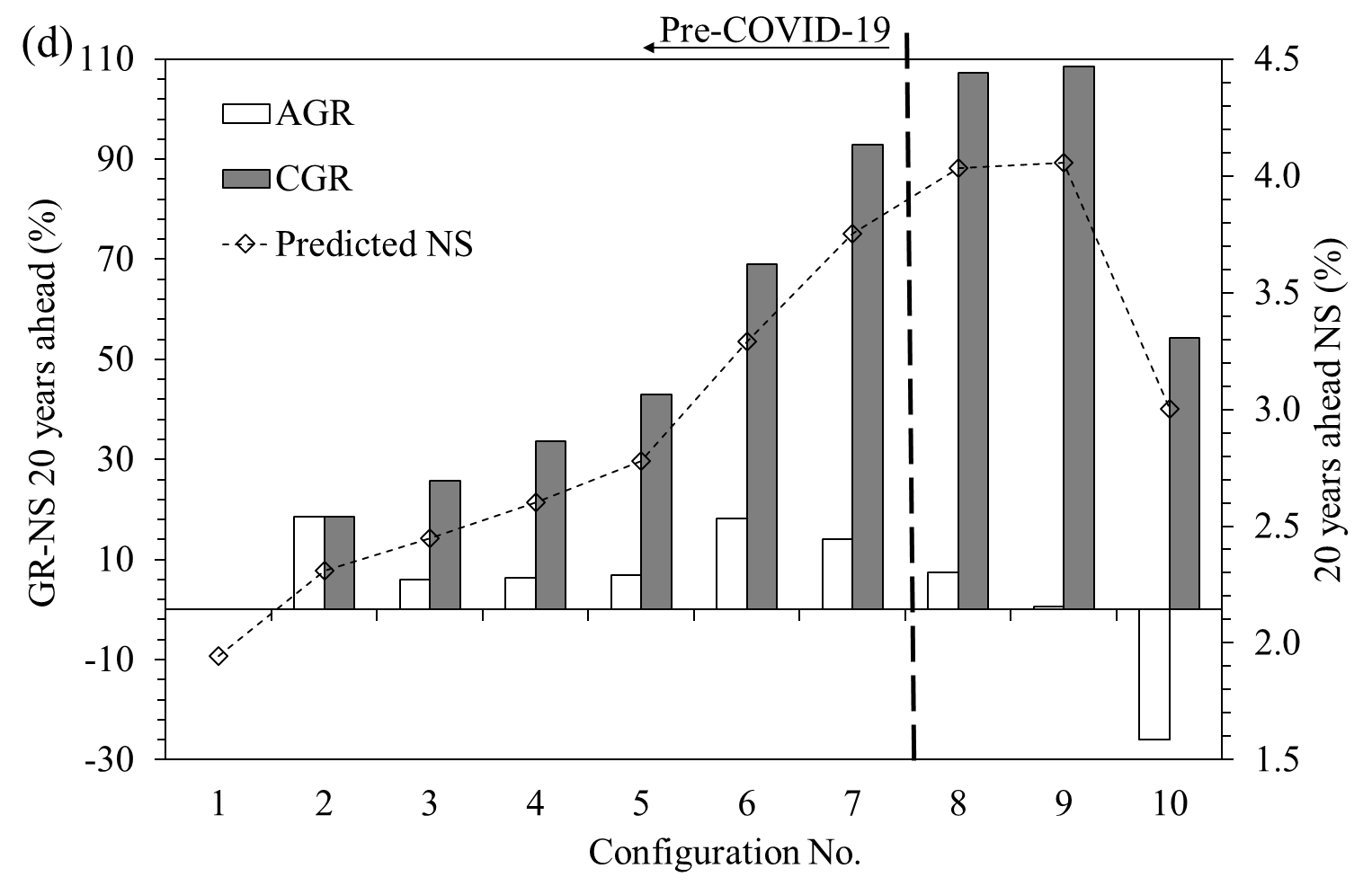

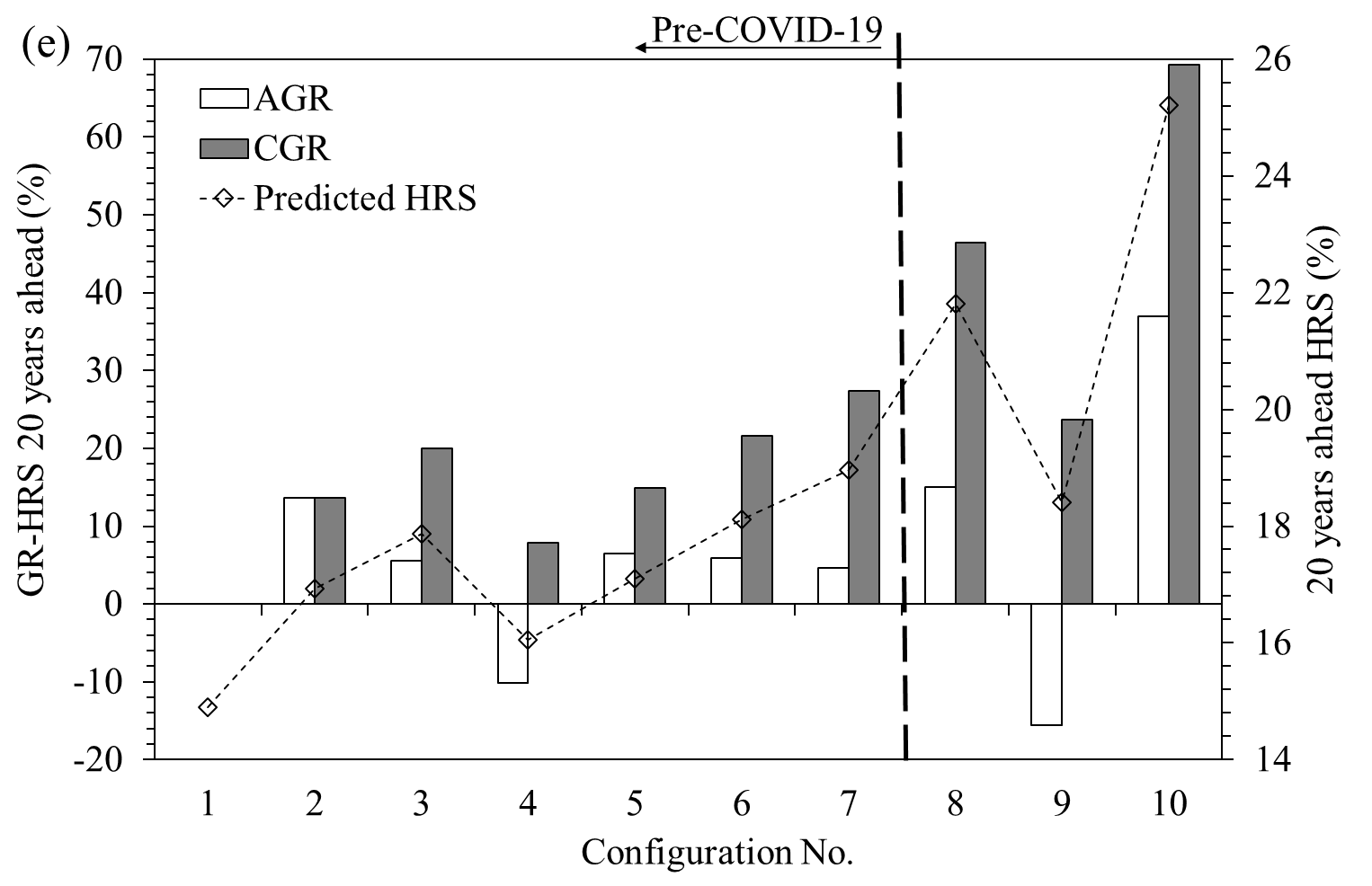


Figure D2. Twenty year ahead predictive evolution of resource shares and their growth rates for: (a) OS (b) GS (c) CS (d) NS (e) HRS.
